# Efficacy of L-Carnitine for Dilated Cardiomyopathy: A Meta-Analysis of Randomized Controlled Trials

**DOI:** 10.1155/2021/9491615

**Published:** 2021-01-12

**Authors:** Yayun Weng, Shuo Zhang, Wei Huang, Xianze Xie, Zhiyuan Ma, Qiaomei Fan

**Affiliations:** ^1^Department of Pharmacy, The First Affiliated Hospital of Zhejiang Chinese Medical University, Hangzhou 310006, China; ^2^Department of Clinical Pharmacology, Key Laboratory of Clinical Cancer Pharmacology and Toxicology Research of Zhejiang Province, Affiliated Hangzhou First People's Hospital, Zhejiang University School of Medicine, Hangzhou, Zhejiang 310006, China

## Abstract

**Background:**

L-carnitine mediates the utilization of fatty acids and glucose in the myocardium. The potential of L-carnitine in managing dilated cardiomyopathy (DCM) in patients has been extensively reported, with additional benefits.

**Objective:**

This meta-analysis purposed to explore the clinical efficacy of L-carnitine therapy on DCM patients.

**Methods:**

We searched publications up to May 2020 from several databases including PubMed, Embase, Cochrane Library, Chinese Biomedical (CBM) database, Chinese Science and Technology Periodicals database (VIP), Chinese National Knowledge Infrastructure (CNKI) database, and Wanfang database. Subsequently, publications that met the inclusion criteria were systematically evaluated by two independent reviewers.

**Results:**

A total of 23 RCTs conducted in China with 1455 DCM patients were included in this study. In the meta-analysis, L-carnitine therapy was associated with a considerable improvement in the overall efficacy (RR = 1.28, 95% CI (1.21-1.36), *P* < 0.0001), left ventricular ejection fraction (LVEF) (MD = 6.16%, 95% CI (4.50, 7.83), *P* < 0.0001), and cardiac output (CO) (MD = 0.88 L/min, 95% CI (0.51, 1.25), *P* < 0.0001) as compared to the control group. Moreover, L-carnitine therapy significantly decreased left ventricular end-diastolic dimension (LVEDD) (MD = −2.53, 95% CI (-3.95, -1.12), *P* = 0.0005), brain natriuretic peptide (BNP) (SMD = −1.71 ng/L, 95% CI (-3.02, -0.40), *P* = 0.01), and the transforming growth factor-beta (TGF-*β*1) (MD = −56.78 ng/L, 95% CI (-66.02, -47.53), *P* < 0.0001).

**Conclusions:**

L-carnitine potentially enhanced the therapeutic efficiency in DCM patients. Following weaknesses in the evidence due to low methodological quality and high clinical heterogeneity in the included studies, well-designed trials are recommended.

## 1. Introduction

Dilated cardiomyopathy (DCM) is among the heterogeneous myocardial disorders characterized by left ventricular dilation and systolic dysfunction in the absence of valvular, congenital, or ischemic heart disease or hypertension [1]. Notably, DCM is a common cause of heart failure, leading to arrhythmias and sudden death [2]. However, the possibilities of idiopathic DCM prevalence are approximately 1 in 250 individuals [3]. Of concern, approximately 10,000 deaths and 46,000 admissions due to DCM were reported in the United States, which equates to an approximate cost of $177 million per year [4]. Elsewhere, an estimate of 12%-20% of the three-year mortality rate was established [5]. More importantly, DCM not only significantly reduces the quality of life in patients but also results in high admission, readmission, and mortality rates accompanied by a financial burden.

There are limited studies on the etiology of DCM. However, DCM treatments focus on improving heart failure, arrhythmias, and conduction system complications [6]. Drugs with angiotensin-converting enzyme (ACE) inhibitors, angiotensin II receptor antagonists (ARBs), *β*-blockers, aldosterone antagonists, diuretics, or digoxin have been adopted in conventional management of DCM [7]. Despite the advances in treatments over the past few decades, the survival rate and overall prognosis of patients need improvements. There are possible benefits of adding adjuvant therapy to conventional therapy. According to studies, cardiac dysfunction is significantly related to abnormal fatty acid metabolism in myocardial cells, while L-carnitine participates in *β*-oxidation of fatty acids in cardiomyocytes. Supplementation of exogenous L-carnitine potentially stimulates myocardial fatty acid metabolism and improves myocardial function [8]. Plasma L-carnitine levels in DCM patients may be a risk factor for survival. Meanwhile, L-carnitine administration can improve three-year survival in DCM patients [9, 10].

Recently, numerous studies have been conducted to determine the effects of L-carnitine treatment in DCM patients [10]. Consequently, we performed a meta-analysis to evaluate the RCT results that investigate the efficacy of L-carnitine combined with conventional therapy in DCM patients.

## 2. Methods

This meta-analysis was performed under Preferred Reporting Items for Systematic Reviews and Meta-Analyses (PRISMA) statement, and the research protocol was registered on PROSPERO (CRD42020161810).

### 2.1. Search Strategy

Publications were obtained following systematic searches on major electronic databases including PubMed, Embase, Cochrane Library, Chinese Biomedical (CBM) database, Chinese Science and Technology Periodicals database (VIP), Chinese National Knowledge Infrastructure (CNKI) database, and Wanfang database. The search period was up to May 2020, and the following keywords were used: “Dilated Cardiomyopathy,” “L-Carnitine,” “Carnitine,” “Levocarnitine,” “Vitamin BT,” and “Bicarnesine.” The search was limited to human subjects with no restriction of languages. The detailed search strategy can be accessed in Supplementary Materials (see Table S1).

### 2.2. Inclusion Criteria and Exclusion Criteria

Two independent reviewers screened the studies based on the following items and discuss the emerging inconsistencies.

#### 2.2.1. Inclusion Criteria

Studies were selected based on the following criteria: (1) randomized controlled trials (RCTs); (2) patients diagnosed with DCM based on the diagnostic criteria followed by their acceptance criteria established by WHO/ISFC [1], European society of cardiology (ESC) [2], and Chinese textbooks [11–14]. The cardiac function of the patients is classified as grades II to IV, according to the New York Heart Association (NYHA) classification. (3) For the included RCTs, the control group was treated with conventional therapy (such as ACE inhibitors, ARBs, *β*-blockers, aldosterone antagonists, diuretics, or digoxin). The experimental group was treated with L-carnitine in addition to the conventional therapy of the control group. (4) Outcomes of (a) overall efficacy: according to NYHA classification, patients who upgrade their class after treatment were regarded effective, (b) left ventricular ejection fraction (LVEF), (c) left ventricular end-diastolic diameter (LVEDD), (d) cardiac output (CO), (e) brain natriuretic peptide (BNP) levels, and (f) transforming growth factor-beta (TGF-*β*1) levels.

#### 2.2.2. Exclusion Criteria

Exclusion criteria included the following items: (1) trials with unclear diagnostic criteria, (2) trials whose allocation methods use the date of birth or date of admission, and (3) trials not mentioning the dose or course of L-carnitine. The experimental group are those receiving drugs other than L-carnitine, on the basis of the treatment of the control group.

### 2.3. Data Extraction

Two independent reviewers extracted data using a standardized data extraction form based on the first author, year of publication, sample size, age, diagnosis standard, NYHA classification, pharmacotherapy intervention, dosage, duration, and outcomes. The disagreement was resolved through concessions.

### 2.4. Quality Assessment

The quality of RCTs included was determined according to the Cochrane Risk of Bias Tool with due consideration of the following domains: random sequence generation, allocation concealment, blinding of participants and personnel, blinding of outcome assessors, incomplete outcome data, selective reporting, and other bias. Two reviewers independently assessed the quality, and differences were resolved through a consensus with the third reviewer.

### 2.5. Statistical Analysis

Meta-analyses of outcomes were performed using Review Manager 5.3 software. 95% confidence intervals (CIs) were used in calculating RR, MD, or SMD for comparing dichotomous and continuous variables, respectively. The degree of heterogeneity among trials was evaluated and quantified by Cochran's *Q* test and *I*^2^. Data with low heterogeneity (*P* ≥ 0.10 and *I*^2^ ≤ 50%) were assessed with a fixed effects model. Similarly, data with high heterogeneity (*P* < 0.10 and *I*^2^ > 50%) was assessed with a random effects model when clinical heterogeneity was excluded. We analyzed the heterogeneity sources to determine the need for either subgroup or sensitivity analyses. Furthermore, analysis for heterogeneity sources may determine the use of a random effects model.

## 3. Results

### 3.1. Search Results

**Figure 1:**
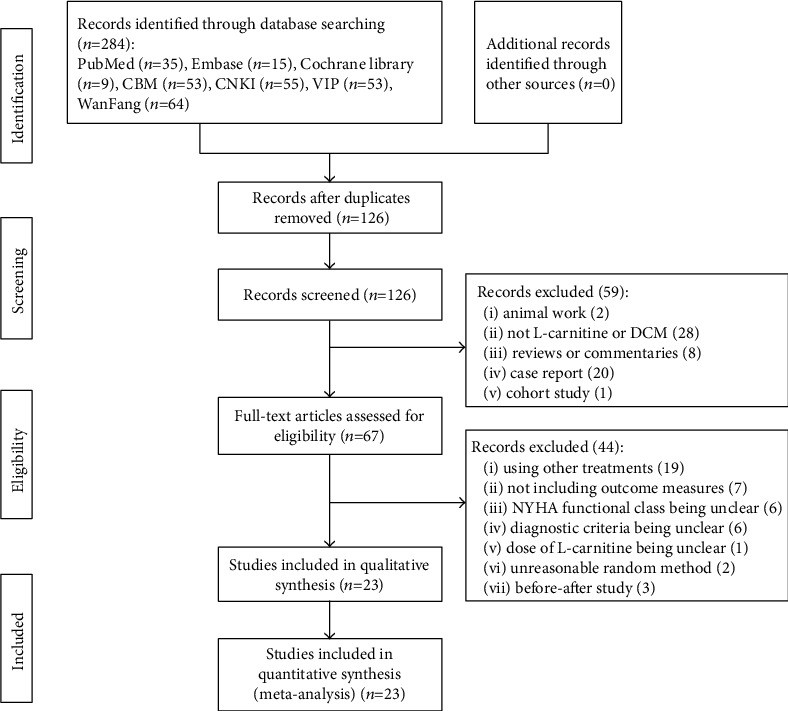
Search results and flow chart of study selection for meta-analysis.

From the initial search, 284 studies were identified. After screening titles and abstracts, 67 studies were eligible for full-text review, of which 23 RCTs met all inclusion criteria. The screening process is summarized in the study flowchart (Figure 1).

### 3.2. Study Characteristics

This analysis had 23 RCTs with 1455 DCM patients. All studies were published between 2006 and 2019. Sample sizes of the study ranged from 29 to 120 patients. All the RCTs included were conducted in China, with 1 study published in English language [15] and the others published in Chinese language. The NYHA classification of patients was between II and IV. A total of 3 studies had children [15–17] on an oral L-carnitine dose (50-100 mg/kg) daily for 1 year, or by intravenous injection for 14 days. Patients in 20 studies were adults [18–37] whereby L-carnitine was administered through intravenous injection or changed to oral administration after injection. The L-carnitine dose ranged from 1 to 6 g per day for 10 to 28 days by intravenous injection or by intravenous injection for 14 days then changes to oral administration for 2 to 6 months. Characteristics of the included RCTs are shown in Table 1.

### 3.3. Quality Assessment

**Figure 2:**
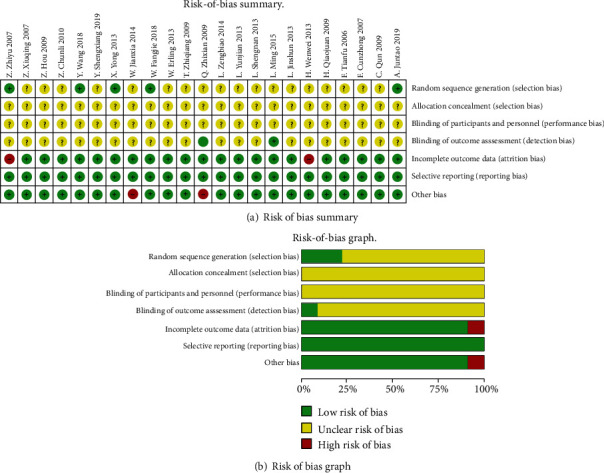
Risk of bias assessment.

In all the RCT studies, five reported sequence generation methods based on the random number table method [16, 18, 33, 34] and simple randomization method [15], respectively. None of the studies either described allocation concealment or used placebo controls. However, two studies [25, 29] mentioned blinding. Dropouts were on two studies [23, 34]. Moreover, two studies [29, 32] had no comparison on the baseline characteristics of participants, which resulted in a high risk of bias to other bias (see Table S2). The results of the assessment of the risk of bias are presented in Figures 2(a) and 2(b).

### 3.4. Meta-analysis

#### 3.4.1. Overall Efficacy according to NYHA Classification

**Figure 3:**
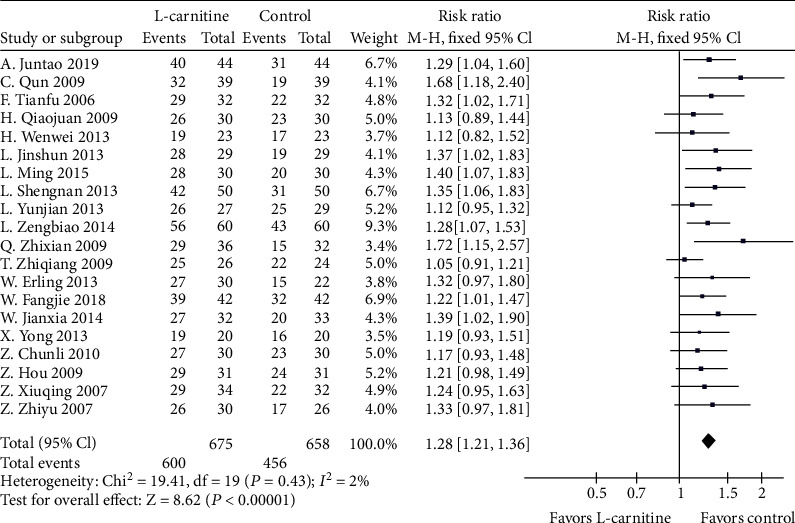
Forest plot of overall efficacy.

According to NYHA classification, patients who upgraded their class after treatment were regarded as effective. However, patients with deterioration or no improvement following treatment application were regarded noneffective. A total of 20 studies [16, 18, 19, 21–35, 37] provided analyzable data for overall efficacy. A fixed effects model was performed because of low heterogeneity (*P* = 0.43, *I*^2^ = 2%). Meta-analysis showed a significant improvement in overall efficacy (RR = 1.28, 95% CI (1.21-1.36), *P* < 0.0001) of patients who received L-carnitine therapy as compared with the controls. The results are shown in Figure 3.

#### 3.4.2. LVEF, LVEDD, and CO

**Figure 4:**
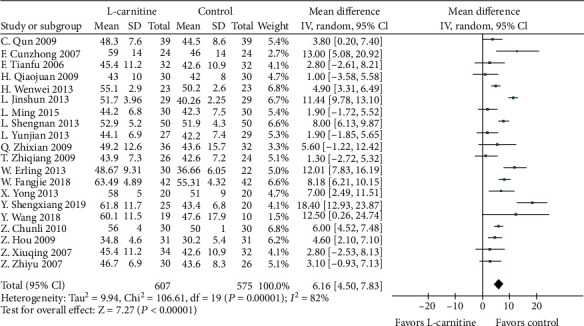
Forest plot of improvement of patients' LVEF.

**Figure 5:**
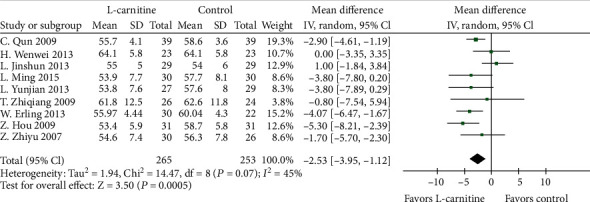
Forest plot of improvement of patients' LVEDD.

**Figure 6:**
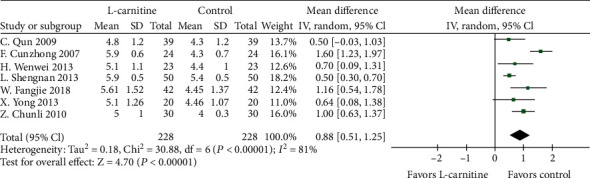
Forest plot of improvement of patients' CO.

A total of 20 studies [15–17, 19–27, 29–31, 33–37] were used to determine the effect of L-carnitine on improving LVEF. A random effects model was performed following the presence of significant heterogeneity (*P* < 0.001, *I*^2^ = 82%) (Figure 4). Meta-analysis showed that LVEF was significantly increased in patients who received L-carnitine therapy than in the control groups (MD = 6.16%, 95% CI (4.50, 7.83), *P* < 0.0001). Indeed, L-carnitine was associated with a significant drop in LVEDD for patients in 9 studies [19, 23–25, 27, 30, 31, 34, 37] (MD = −2.53, 95% CI (-3.95, -1.12), *P* = 0.0005) (Figure 5). This outcome was analyzed with a random effects model as there was significant heterogeneity among the studies (*P* = 0.07, *I*^2^ = 45%). A sensitivity analysis was performed by removing the study by L. Jinshun. The heterogeneity significantly decreased while the result was consistent with the primary analysis. Meta-analysis shows that CO was significantly higher in patients who received L-carnitine therapy in 7 studies [16, 19, 20, 23, 26, 33, 36] than the control group (MD = 0.88 L/min, 95% CI (0.51, 1.25), *P* < 0.0001) (Figure 6). Heterogeneity was significant among the studies when comparing CO (*P* < 0.001, *I*^2^ = 81%). Thus, a random effects model was used.

#### 3.4.3. BNP and TGF-*β*1

**Figure 7:**
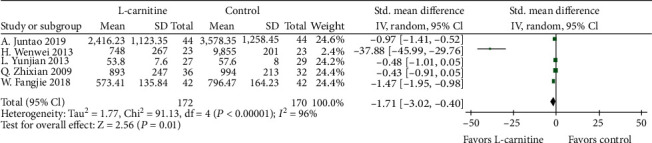
Forest plot of decrease in patients' BNP.

**Figure 8:**
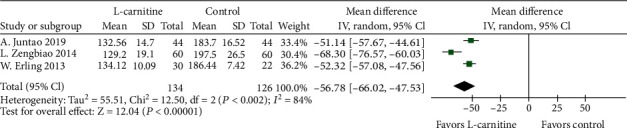
Forest plot of decrease in patients' TGF-*β*1.

A total of 5 studies [16, 18, 23, 27, 29] were used to assess the effect of L-carnitine with a decreasing BNP. The results were presented as SMD due to multiple measurement methods and a large difference in the data mean. However, the meta-analysis suggested that L-carnitine therapy significantly reduces BNP levels (SMD = −1.71 ng/L, 95% CI (-3.02, -0.40), *P* = 0.01) as compared to the control group (Figure 7). There was a significant heterogeneity among the studies (*P* < 0.001, *I*^2^ = 96%). Following the sensitivity analysis after removing the study of H. Wenwei [23], the result was consistent with the initial analysis. Among the 3 studies [18, 28, 31] that compared levels of TGF-*β*1, their meta-analysis showed a significant decrease in patients receiving L-carnitine (MD = −56.78 ng/L, 95% CI (-66.02, -47.53), *P* < 0.0001) (Figure 8). High heterogeneity was found among the studies (*P* = 0.002, *I*^2^ = 84%).

### 3.5. Publication Bias

**Figure 9:**
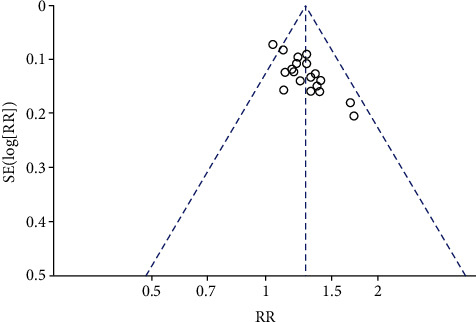
Bias funnel plot of publication.

The funnel plot was used to evaluate the publication biases in the meta-analysis to determine the overall efficacy according to NYHA classification (Figure 9). Since the included studies were mostly from China, we hypothesize that the publication bias might exist from this analysis.

## 4. Discussion

This review examined 23 RCTs that assessed the effectiveness of L-carnitine in the treatment of DCM patients. Our meta-analysis showed that L-carnitine increased LVEF, CO, and overall efficacy and decreased LVEDD, BNP, and TGF-*β*1 of DCM patients.

LVEF is a widely adopted parameter of systolic dysfunction for assessment. A wealth of studies has associated reworsening of LVEF with poor cardiac outcomes [38–40]. CO results from the comprehensive factors from left ventricular preload and afterload [41]. Elsewhere, a persistently high BNP level was established as a strong predictor of death, transplant, or hospitalization for DCM patients [42]. More importantly, TGF-*β*1 plays an important role in myocardial cell hypertrophy and cardiac interstitial growth [43]. Besides, DCM is associated with raised levels of TGF-*β*1 [44].

Our review revealed that L-carnitine represents an effective adjuvant therapy that potentially ameliorates clinical symptom and cardiac function in DCM patients. The addition of L-carnitine to the conventional treatment for DCM patients has promising potential in achieving clinical benefits.

Notably, from the efficacy study of L-carnitine on the mortality rate in DCM patients, there was an improvement in their mortality rate on L-carnitine patients for 3 years of follow-up data (18% placebo group vs. 3% L-carnitine group) [9]. A study by El-Aroussy et al. [45] showed a parallel correlation between plasma and urinary L-carnitine levels with left ventricular function as determined by echocardiography. Both Azevedo et al. [46] and Kotby et al. [47] in their cohort and before-after studies demonstrated that the LVEF was increased in L-carnitine-treated patients. Thus, these studies revealed that L-carnitine has a superior performance in clinical efficiency of DCM though they were not included in our study following inconsistencies in outcome indicators or the study types.

L-carnitine mediates the utilization of fatty acids and glucose in the myocardium. Studies have demonstrated that changes in fatty acid utilization and energy metabolism disorders in myocardial cells lead to myocardial structural and functional abnormalities, which will progress into heart failure. The heart is largely dependent on fatty acid oxidation as a source of energy. Changes in fatty acid utilization and energy metabolism disorders in myocardial cells lead to myocardial structural and functional abnormalities. Insufficient ATP supply comprises one of the major reasons governing left ventricular damage [48]. L-carnitine is an important cofactor that mediates the oxidation of long-chain fatty acids into the mitochondrial membrane which subsequently promotes the oxidation of fatty acids, hence improving the level of ATP in cardiomyocytes [49]. L-carnitine improves energy metabolism in cardiomyocytes, which alleviated myocardial damage and protected cardiac function. Furthermore, L-carnitine exerts a cardioprotective effect using alternative mechanisms such as suppressing cardiac fibrosis, nitric oxide production, or interstitial remodeling [50, 51]. Previous studies have determined that L-carnitine concentration decreased in cardiac tissue of DCM patients as compared to those of healthy people [52]. There are beneficial effects of L-carnitine in congestive heart failure [39]. However, our research showed that the addition of L-carnitine to the treatment of DCM patients may have an additional improvement in heart functioning.

There were some limitations in the methodological qualities of our included studies. For instance, only 5 of the total trials reported how the participants were randomly assigned. None of the trials mentioned the use of allocation concealment. There were only two trials that used blind methods and another two trials that reported withdrawal and loss of follow-up. Therefore, excluding the potential risk of bias was nearly impossible. Besides, the follow-up duration varied widely from 10 days to 1 year, which led to significant heterogeneity among the trials. Furthermore, the trials included were mainly conducted in China; thus, we hypothesized that publication bias might exist in the meta-analysis.

## 5. Conclusions

Herein, the present study demonstrated that L-carnitine could be used as an effective therapy in DCM patients, which could improve cardiac functioning. However, the small sample size, poor methodological quality, and high clinical heterogeneity from the included study weaken the results. Therefore, well-designed trials with large sample size are recommended in future.

## Figures and Tables

**Table 1:** Characteristics of included RCTs.

Study	Sample size (T/C)	Age (years)	Diagnosis standard	NYHA classification	Intervention	Control	Course	Outcomes
A. Juntao 2019	44/44	T: 60.0 ± 11.0 C: 60.8 ± 10.8	Chinese publications	II-IV	L-carnitine, 3.0 g/d, iv.+CVT	CVT	14 days	Overall efficacy, BNP, TGF-*β*1
C. Qun 2009	39/39	T: 66.8 ± 7.6 C: 67.6 ± 6.2	WHO/ISFC	III-IV	L-carnitine, 4.0 g/d, iv.+CVT	CVT	20 days	Overall efficacy, LVEF, LVEDD, CO
F. Cunzhong 2007	24/24	30-68	Chinese publications	II-IV	L-carnitine, 1.0 g/d, iv.+CVT	CVT	21 days	Overall efficacy, LVEF, CO
F. Tianfu 2006	32/32	T: 42-78 C: 39-77	WHO/ISFC	II-IV	L-carnitine, 2.0 g/d, iv.+CVT	CVT	10 days	Overall efficacy, LVEF
H. Qiaojuan 2009	30/30	T: 50 ± 15 C: 51 ± 17	WHO/ISFC	II-IV	L-carnitine, 3.0 g/d, iv.+CVT	CVT	21 days	Overall efficacy, LVEF
H. Wenwei 2013	23/23	57.4 ± 2.1	WHO/ISFC	II-IV	L-carnitine, 3.0 g/d, iv. then 1.0 g/d, po.+CVT	CVT	iv. 14 days, then po. 2 months	Effective rate, LVEF, LVEDD, CO, BNP
L. Jinshun 2013	29/29	35-58	Chinese publications	II-III	L-carnitine, 2.0 g/d, iv.+CVT	CVT	14 days	Overall efficacy, LVEF, LVEDD
L. Ming 2015	30/30	53.4 ± 6.8	WHO/ISFC	III-IV	L-carnitine, 3.0 g/d, iv.+CVT	CVT	14 days	Overall efficacy, LVEF, LVEDD
L. Shengnan 2013	50/50	T: 65.4 ± 7.4 C: 63.2 ± 6.9	WHO/ISFC	III-IV	L-carnitine, 4.0 g/d, iv.+CVT	CVT	14 days	Overall efficacy, LVEF, CO
L. Yunjian 2013	27/29	T: 62.8 ± 8.8 C: 54.1 ± 6.9	WHO/ISFC	III-IV	L-carnitine, 3.0 g/d, iv.+CVT	CVT	14 days	Overall efficacy, LVEF, LVEDD, BNP
L. Zengbiao 2014	60/60	T: 62.8 ± 8.8 C: 63.2 ± 8.3	WHO/ISFC	II-IV	L-carnitine, 2.0 g/d, iv.+CVT	CVT	14 days	Overall efficacy, TGF-*β*1
Q. Zhixian 2009	36/32	52.8 ± 13.7	Chinese publications	III-IV	L-carnitine, 2.0 g/d, iv.+CVT	CVT	14 days	Overall efficacy, LVEF, BNP
T. Zhiqiang 2009	26/24	T: 55.5 ± 8.3 C: 54.2 ± 8.1	WHO/ISFC	III-IV	L-carnitine, 3.0 g/d, iv. then 3.0 g/d, po.+CVT	CVT	iv. 14 days, then po. 6 months	Overall efficacy, LVEF, LVEDD
W. Erling 2013	30/22	T: 63.6 ± 8.2 C: 62.7 ± 8.6	WHO/ISFC	III-IV	L-carnitine, 2.0 g/d, iv.+CVT	CVT	14 days	Overall efficacy, LVEF, LVEDD, TGF-*β*1
W. Jianxia 2014	32/33	55-75	WHO/ISFC	II-III	L-carnitine, 3.0 g/d, iv.+CVT	CVT	12 days	Overall efficacy
X. Yong 2013	20/20	NR	WHO/ISFC	II-IV	L-carnitine, 1.0 g/d, iv.+CVT	CVT	28 days	Overall efficacy, LVEF, CO
Z. Zhiyu 2007	30/26	42.6 ± 11.1	WHO/ISFC	II-IV	L-carnitine, 3.0 g/d, iv.+CVT	CVT	21 days	Overall efficacy, LVEF, LVEDD
Z. Xiuqing 2007	34/32	T: 70-85 C: 81-83	WHO/ISFC	II-IV	L-carnitine, 6.0 g/d, iv.+CVT	CVT	10 days	Overall efficacy, LVEF
Z. Chunli 2010	30/30	30-63	WHO/ISFC	II-IV	L-carnitine, 2.0 g/d, iv.+CVT	CVT	28 days	Overall efficacy, LVEF, CO
Z. Hou 2009	31/31	48.8 ± 10.1	Chinese publications	II-IV	L-carnitine, 3.0 g/d, iv.+CVT	CVT	14 days	Overall efficacy, LVEF, LVEDD
W. Fangjie 2018	42/42	T: 4.0 ± 1.0 C: 4.0 ± 1.0	ESC	II-IV	L-carnitine, 100 mg/(kg·d), iv.+CVT	CVT	14 days	Overall efficacy, LVEF, CO, BNP
Y. Shengxiang 2019	25/20	1 month-13 years	WHO/ISFC	II-IV	L-carnitine, 50-100 mg/(kg·d), po.+CVT	CVT	1 year	LVEF
Y. Wang 2018	19/10	1 month-13 years	WHO/ISFC	III-IV	L-carnitine, 50-100 mg/(kg·d), po.+CVT	CVT	1 year	LVEF

Note: CVT: conventional treatment (such as ACE inhibitors, ARBs, *β*-blockers, aldosterone antagonists, diuretics, or digoxin); NR: not reported; T: experimental group; C: control group.

## Data Availability

The data used to support the findings of this study are available from the corresponding author upon request.

## Supplementary Materials

Supplementary materials Table S1: search strategy in PubMed. Table S2: quality assessment of randomized studies.
