# Supplementary material for: Efficacy of L-Carnitine for Dilated Cardiomyopathy: A Meta-Analysis of Randomized Controlled Trials
Source: Biomed Res Int. 2021 Jan 12;2021:9491615. doi: 10.1155/2021/9491615 (PMC7817303; doi:10.1155/2021/9491615)
Supplement: Supplementary materials — Table S1: search strategy in PubMed. Table S2: quality assessment of randomized studies. [file 9491615.f1.doc]

**Efficacy of L-Carnitine for Dilated Cardiomyopathy: A Meta-Analysis of** **Randomized Controlled Trials**

**Supplementary Material**

**Table S1. Search strategy in PubMed**

**Table S2. Quality assessment of randomized studies**

**Table S1. Search strategy in PubMed**

| **Step** | **Search term** | **Results** |
| --- | --- | --- |
| 1 | "Cardiomyopathy, Dilated"[Mesh] | 15,614 |
| 2 | (Cardiomyopathy, Dilated) OR (Cardiomyopathies, Dilated) OR (Dilated Cardiomyopathy) OR (Cardiomyopathy, Familial Idiopathic) OR (Familial Idiopathic Cardiomyopathy) OR (Cardiomyopath*, Congestive) OR (Congestive Cardiomyopath*) OR (Cardiomyopathy, Idiopathic Dilated) OR (Dilated Cardiomyopathy, Idiopathic) OR (Idiopathic Dilated Cardiomyopath*) | 27,015 |
| 3 | #1 OR #2 | 27,015 |
| 4 | "Carnitine"[Mesh] | 9,657 |
| 5 | Carnitine OR Levocarnitine OR Vitamin BT OR L-Carnitine OR (L Carnitine) OR Bicarnesine | 17,878 |
| 6 | #4 OR #5 | 17,878 |
| 7 | #3 AND #6 | 167 |
| 8 | (Randomized controlled trial [pt]) OR (Controlled clinical trial [pt]) OR Randomized [tiab] OR Placebo [tiab] OR (Clinical trials as topic [mesh: noexp]) OR Randomly [tiab] OR (Drug therapy[sh]) OR Trial [tiab] | 3,325,906 |
| 9 | #7 AND #8 | 47 |
| 10 | "Humans"[mh] | 18,525,878 |
| 11 | #9 AND #10 | 35 |

**Table S2. Quality assessment of randomized studies**

| **No** | **Author, year** | **Random sequence generation** | **Allocation concealment** | **Blinding (patient)** | **Blinding (assessor)** | **Incomplete outcome data** | **Selective reporting** | **Other bias** |
| --- | --- | --- | --- | --- | --- | --- | --- | --- |
| 1 | A. Juntao 2019 | Low (random number table) | Unclear  (no information) | Unclear  (no information) | Unclear  (no information) | Low  (no dropout) | Low (no missing outcomes) | Low |
| 2 | C. Qun 2009 | Unclear  (no information) | Unclear  (no information) | Unclear  (no information) | Unclear  (no information) | Low  (no dropout) | Low (no missing outcomes) | Low |
| 3 | F. Cunzhong 2007 | Unclear  (no information) | Unclear  (no information) | Unclear  (no information) | Unclear  (no information) | Low  (no dropout) | Low (no missing outcomes) | Low |
| 4 | F. Tianfu 2006 | Unclear  (no information) | Unclear  (no information) | Unclear  (no information) | Unclear  (no information) | Low  (no dropout) | Low (no missing outcomes) | Low |
| 5 | H. Qiaojuan 2009 | Unclear  (no information) | Unclear  (no information) | Unclear  (no information) | Unclear  (no information) | Low  (no dropout) | Low (no missing outcomes) | Low |
| 6 | H. Wenwei 2013 | Unclear  (no information) | Unclear (no information) | Unclear  (no information) | Unclear  (no information) | High  (3 dropout) | Low (no missing outcomes) | Low |
| 7 | L. Jinshun 2013 | Unclear  (no information) | Unclear  (no information) | Unclear  (no information) | Unclear  (no information) | Low  (no dropout) | Low (no missing outcomes) | Low |
| 8 | L. Ming 2015 | Unclear  (no information) | Unclear  (no information) | Unclear  (no information) | Low  (blinded) | Low  (no dropout) | Low (no missing outcomes) | Low |
| 9 | L. Shengnan 2013 | Unclear  (no information) | Unclear  (no information) | Unclear  (no information) | Unclear  (no information) | Low  (no dropout) | Low (no missing outcomes) | Low |
| 10 | L. Yunjian 2013 | Unclear  (no information) | Unclear  (no information) | Unclear  (no information) | Unclear  (no information) | Low  (no dropout) | Low (no missing outcomes) | Low |
| 11 | L. Zengbiao 2014 | Unclear  (no information) | Unclear  (no information) | Unclear  (no information) | Unclear  (no information) | Low (no dropout) | Low (no missing outcomes) | Low |
| 12 | Q. Zhixian 2009 | Unclear  (no information) | Unclear  (no information) | Unclear  (no information) | Low  (blinded) | Low  (no dropout) | Low (no missing outcomes) | High (no comparison on the baseline characteristics of participants) |
| 13 | T. Zhiqiang 2009 | Unclear  (no information) | Unclear  (no information) | Unclear  (no information) | Unclear  (no information) | Low  (no dropout) | Low (no missing outcomes) | Low |
| 14 | W. Erling 2013 | Unclear  (no information) | Unclear  (no information) | Unclear  (no information) | Unclear  (no information) | Low  (no dropout) | Low (no missing outcomes) | Low |
| 15 | W. Jianxia 2014 | Unclear  (no information) | Unclear  (no information) | Unclear  (no information) | Unclear  (no information) | Low  (no dropout) | Low (no missing outcomes) | High (no comparison on the baseline characteristics of participants) |
| 16 | X. Yong 2013 | Low (random number table) | Unclear  (no information) | Unclear  (no information) | Unclear  (no information) | Low  (no dropout) | Low (no missing outcomes) | Low |
| 17 | Z. Zhiyu 2007 | Low (random number table) | Unclear  (no information) | Unclear  (no information) | Unclear  (no information) | High  (4 dropout) | Low (no missing outcomes) | Low |
| 18 | Z. Xiuqing 2007 | Unclear  (no information) | Unclear  (no information) | Unclear  (no information) | Unclear  (no information) | Low  (no dropout) | Low (no missing outcomes) | Low |
| 19 | Z. Chunli 2010 | Unclear  (no information) | Unclear  (no information) | Unclear  (no information) | Unclear  (no information) | Low  (no dropout) | Low (no missing outcomes) | Low |
| 20 | Z. Hou 2009 | Unclear  (no information) | Unclear  (no information) | Unclear  (no information) | Unclear  (no information) | Low  (no dropout) | Low (no missing outcomes) | Low |
| 21 | W. Fangjie 2018 | Low (random number table) | Unclear  (no information) | Unclear  (no information) | Unclear  (no information) | Low  (no dropout) | Low (no missing outcomes) | Low |
| 22 | Y. Shengxiang 2019 | Unclear  (no information) | Unclear  (no information) | Unclear  (no information) | Unclear  (no information) | Low  (no dropout) | Low (no missing outcomes) | Low |
| 23 | Y. Wang 2018 | Low (simple randomization) | Unclear  (no information) | Unclear  (no information) | Unclear  (no information) | Low  (no dropout) | Low (no missing outcomes) | Low |
